# Synthesis, Characterization, and Sludge Dewaterability Evaluation of the Chitosan-Based Flocculant CCPAD

**DOI:** 10.3390/polym11010095

**Published:** 2019-01-08

**Authors:** Chunhong Shi, Wenquan Sun, Yongjun Sun, Lei Chen, Yanhua Xu, Mengdan Tang

**Affiliations:** 1 School of Energy and Environmental Engineering, University of Science and Technology Bejing, Bejing 100083, China; sch.22@163.com; 2 College of Urban Construction, Nanjing Tech University, Nanjing 211800, China; 18251857615@126.com; 3 Jiangsu Key Laboratory of Industrial Water-Conservation & Emission Reduction, College of Environment, Nanjing Tech University, Nanjing 211800, China; matlabvisual@163.com (L.C.); yanhuaxu18@hotmail.com (Y.X.)

**Keywords:** chitosan-based flocculant, graft copolymerization, flocculation, sludge conditioning, dewaterability

## Abstract

Carboxymethyl chitosan (CMCS), acrylamide, and methacryloxyethyltrimethyl ammonium chloride were used as co-monomers to produce a sludge dewatering agent carboxymethyl chitosan-graft-poly(acrylamide-methacryloxyethyltrimethyl ammonium chloride) (CCPAD) by UV-induced graft polymerization. Single-factor experiments and response surface methodology were employed to investigate and optimize the grafting rate, grafting efficiency, and intrinsic viscosity influenced by the total monomer concentration, CMCS concentration, cationic degree, pH value, and illumination time. The structure, surface morphology, and thermal stability of CCPAD were characterized by infrared spectroscopy, hydrogen nuclear magnetic resonance, X-ray diffraction, scanning electron microscopy, and differential thermal-thermogravimetry. The raw sludge with 97.9% water content was sourced from the concentrated tank of a sewage treatment plant and used in the sludge condition experiments. In addition, CCPAD was applied as the sludge conditioner to investigate the effects of cationic degree, intrinsic viscosity, and pH on the supernatant turbidity, moisture content, specific resistance to filtration, and sludge settling ratio. Moreover, the mechanism of sludge conditioning by CCPAD was discussed by examining the zeta potential and extracellular polymeric substance (EPS) content of the supernatant. The sludge dewatering results confirmed that CCPAD had excellent performance for improving sludge dewaterability.

## 1. Introduction

Municipal sewage treatment plant sludge has high organic content, unstable nature, fine particles, large specific surface area, and negative charge [1]. This substance can also easily form a dispersion system with poor settling properties. In addition, sludge composition markedly varies depending on the source of the sewage, the sewage treatment process, and the season [2]. As a by-product of wastewater treatment, the sludge also contains pathogenic bacteria, parasite eggs, heavy metals, and other substances. Thus, the sludge contains heavy metals and toxic substances that easily pass through the food chain and eventually enter the human body with safety risks [3]. Improperly handled sludge would cause serious secondary pollution. The treatment and disposal of sludge have become an important step in sewage treatment such that sludge treatment is also an important standard for evaluating the quality of sewage treatment [4]. The disposal of sludge has certain requirements for the moisture content of the sludge [5]. To reduce, stabilize, remove the harmfulness of the sludge treatment, and comprehensively utilize the resources, sludge is usually flocculated, conditioned, and dewatered. This process reduces the moisture content of the sludge and the sludge volume and facilitates the transport and subsequent processing to meet the moisture content requirements [6].

Sludge dewatering is a crucial step in sludge treatment. The effect of this process is closely related to the sludge treatment system and influences subsequent disposal [7]. However, due to the high moisture content of raw sludge, the particles in the sludge are small and negatively charged with strong hydrophilicity [8]. The water molecule and sludge particles are closely connected such that the sludge particles form a stable colloidal suspension [9]. This characteristic hinders the separation of mud and water, and the performance of direct sludge dewatering is poor [10]. Therefore, sludge conditioning before sludge dewatering is particularly important. Conditioning can change the physicochemical properties of the sludge and increase cohesion, the size of the particles, and the efficiency of sludge dewatering [11]. At present, sludge conditioning includes physical, chemical, and microbial conditioning [12]. However, physical conditioning can increase the capacity expansion ratio of the sludge (the volume ratio before and after sludge sludge dewatering), which leads to additional costs to the disposal of this substance [13]. Microbial preparation entails high cost, long culture period of strains, and a difficult stabilization process. Thus, the application range of this approach is limited. Chemical conditioning methods are widely used in sludge dewatering because of their simple operation, low investment cost, and stable conditioning performance [14]. Organic flocculants, such as anionic and cationic flocculants, are widely used among these chemical conditioners [15]. Compared with anionic flocculants, cationic polymer flocculants have a high sedimentation rate, good electrical neutralization capacity, low dosage, wide application range, and good sludge dewatering performance with some extent of acid and alkali resistance [16].

As a natural polymer material, chitosan is a cationic polyelectrolyte because of the protonation of amino groups and has been used in many fields [17]. However, its application for preparing flocculants is restricted because of its solubility in acidic solutions [18]. Carboxymethyl chitosan (CMCS) is obtained by introducing carboxymethyl to chitosan. Thus, CMCS molecular chains contain –COOH, –NH_2_, –OH, and other groups with special physical and chemical capacity [19]. The CMCS can be modified using graft copolymerization to further improve the flocculation performance of CMCS. The modification is performed to increase the charge density and molecular weight and to further enhance the solubility. Thus, the modified polymer flocculation has enhanced efficiency and sludge dewatering performance [20]. In addition, the graft copolymerization of CMCS with cationic monomers combines the advantages of cationic, anionic, and other functional groups. These groups can remarkably increase the charge density and adsorb-bridging ability. Thus, the molecular chain length is enhanced, and the scope of application is gradually extended [21]. However, few studies have investigated the application of chitosan-grafted polymer in sludge conditioning and flocculation to improve sludge dewaterability.

The preparation of a cationic sludge-dewatering agent, CCPAD, was systematically studied. The application of CCPAD in sludge dewatering and the mechanism of sludge conditioning were initially explored. Photo-initiated graft polymerization was used to prepare cationic copolymer CCPAD using carboxymethyl chitosan (CMCS), acrylamide (AM), and methacryloxyethyltrimethyl ammonium chloride (DMC) as monomers. Single-factor experiments were performed to investigate the effects of total monomer concentration, CMCS concentration, cationic degree, photoinitiator concentration, pH value, and illumination time on the graft polymerization. The grafting rate, grafting efficiency, and intrinsic viscosity were examined, and the optimal preparation conditions were determined by response surface methodology. The structure, morphology, and thermal stability of the polymer products were characterized by infrared spectroscopy, nuclear magnetic resonance spectroscopy, X-ray diffraction (XRD), scanning electron microscopy (SEM), and thermogravimetric analysis. In addition, the effects of CCPAD dosage, cationicity, intrinsic viscosity, and pH on the sludge dewaterability were investigated by determining the supernatant turbidity, moisture content of filter cake, specific resistance of sludge, and sedimentation rate. The conditioning mechanism was initially discussed by the supernatant zeta potential, extracellular polymeric substance, and microscopic morphology of the sludge.

## 2. Materials and Methods

### 2.1. Materials

The sludge was sourced from the concentrated tank of a sewage treatment plant in Nanjing (China), and the properties of the sludge are shown in Table 1. CMCS, AM, DMC, bovine serum albumin (biochemical reagent), 2,2′-Azobis(2-methylpropionamide)dihydrochloride (V-50), and Coomassie brilliant blue were purchased from Aladdin Reagent Co., Ltd. (Shanghai, China). Phenol, sodium hydroxide, concentrated hydrochloric acid, anhydrous ethanol, sodium chloride, and other reagents were of analytical grade and purchased from Nanjing Shengjian Quanhua Glass Instrument Co., Ltd., Nanjing, China.

### 2.2. Synthesis and Characterization of CCPAM

CMCS (2.0 g), 6.0 g AM, 33.0 mL distilled water, and 2.0 g DMC were weighed into a quartz wide-mouthed bottle according to a certain mass ratio and stirred until the three monomers were completely dissolved. Nitrogen was purged for 30 min to remove oxygen, and then a certain amount of 2,2′-Azobis(2-methylpropionamide)dihydrochloride as a photoinitiator (0.1 wt % relative to total monomers) was added. The sealed quartz bottle was placed in a UV lamp reactor for polymerization. After a certain period of reaction (100 min), the reactor was removed and allowed to stand for 1 h to obtain a milky opaque gel. The graft polymer was purified and dried to obtain the CCPAD powder. The graft copolymer colloid product was sheared into particles with a particle diameter of 1–3 mm and then immersed in an ethanol and acetone solution with a volume ratio of 1:1 to remove unreacted monomers and small molecule polymers. The purified CCPAD was dried in a vacuum oven to obtain a solid CCPAD, and finally the solid CCPAD was ground and passed through a 200-mesh screen to obtain a CCPAD powder. The purity of CCPAD was determined according to Chinese national standards (GBT 31246-2014). The CCPAD powder can be obtained by repeating the above experimental procedure.

**Figure 1:**
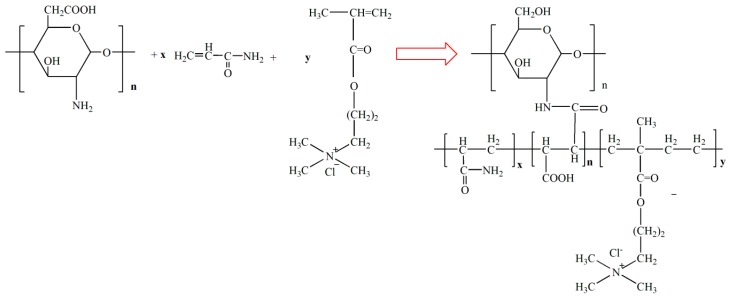
Possible scheme of CCPAD synthesis.

The synthesized CCPAD was characterized using an infrared spectrometer (510PFT-IR, Nicolet, Madison, Wisconsin, USA), nuclear magnetic resonance spectrometer (AVANCE500 Nuclear Magnetic Resonance Apparatus, BRUKER, Ettlingen, Germany), X-ray diffractometer (SmartLabTM 3KW, Japan Science Corporation, Tokyo, Japan), scanning electron microscope (TM3000, HITACHI, Tokyo, Japan), and differential heat-thermogravimetric analyzer (DTG-60H, Shimadzu Corporation, Tokyo, Japan). The cationic degree of CCPAD was determined by colloid titration according to Chinese national standards (GBT 31246-2014). Toluidine blue was used as an indicator, and poly-2-acrylamide-2-methylpropane sulfonate (PAMPSK) was used as a standard polyanion to titrate the cation sample. After the equivalence point, a trace polyanion was combined with toluidine blue to change the solution from blue to purple for indicating the end point. The possible scheme of CCPAD synthesis is shown in Figure 1. As shown in Table 2, compared with other initiation technologies, photoinitiated polymerization can achieve initiation at normal temperature and reduce production energy consumption with no residue in production and lower activation energy. No pollutants were formed in the polymerization process with short polymerization time, simple equipment, and energy saving. Therefore, photoinitiation technology is currently a promising initiation method for synthesizing chitosan-grafted flocculants. 

### 2.3. Sludge Dewatering Tests

The activated sludge was poured into a 1.0 L organic glass beaker and then flocculated by CCPAD with dosage of 0–60 mg/L. After the addition of CCPAD, the sludge was flocculated and conditioned by stirring at 350 rpm for 1 min, followed by 30 min of sedimentation. Moreover, the flocculate sludge was obtained to detect the sludge settling ratio (SV30). The soaked filter paper was put into a Buchner funnel, and the vacuum pump was started to make the filter paper tightly adhere to the funnel. The conditioned sludge was poured into the Buchner funnel to determine the SRF [29]. The filter cake was taken out and dried at 105 °C for 2 h to constant weight to determine the moisture content. The supernatant was then extracted and measured to determine the turbidity of the supernatant, zeta potential, and the extracellular polymer concentration [30].

## 3. Results

### 3.1. Synthesis of CCPAD

The optimization of the synthesis process was systemically investigated through a single-factor experiment (Figures S1–S6). Some key factors were chosen to study the effects of synthetic conditions on the properties of CCPAD. The effects of total monomer concentration, CMCS concentration, cationic degree, photo-initiator concentration, pH, and illumination time on intrinsic viscosity, grafting ratio, and grafting efficiency were investigated and optimized systematically. The optimal synthesis conditions for the grafted polymer CCPAD prepared by photopolymerization were as follows: monomer concentration, 40%; cationic degree, 40%; CMCS concentration, 8%; illumination, 2 h; and pH 8. 

### 3.2. CCPAD Characterization

#### 3.2.1. FTIR Spectra

**Figure 2:**
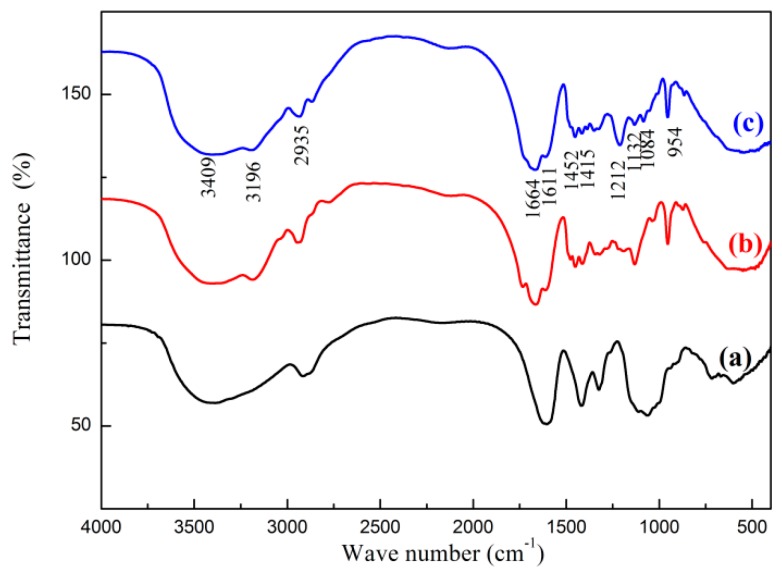
FTIR spectra of (**a**) CMCS, (**b**) P(AM-DMC), and (**c**) CCPAD.

The absorption peaks at 3409 cm^−1^ and 3196 cm^−1^ are attributed to the NH stretching vibration peaks [Figure 2c]. The absorption peak at 2935 cm^−1^ is assigned to the stretching vibration absorption peak of CH in the methyl group and the methylene group of AM, and the stretching vibration absorption peak of C=O is in the region of 1611–1664 cm^−1^ [31]. The absorption peak at 1452 cm^−1^ is attributed to the characteristic absorption peak of methylene on the quaternary ammonium group, and the vibration absorption peak at 1212 cm^−1^ is assigned to the glycosidic bond (COC) on the glycopyran ring of CMCS. The stretching vibration peak at 1132 cm^−1^ is assigned to C–O in C–OH [32]. As shown in Figure 2c, the absorption peak area of N-H stretching vibration at 3409.05 cm^−1^ and 3196.43 cm^−1^ was larger than that in AM and CMCS, which is the superposition of N-H absorption peak in CMCTS and AM. The stretching vibration absorption peak of C=O was observed at 1611.71 cm^−1^–1664.75 cm^−1^, which was produced by superimposing C=O absorption peaks in CMCTS and AM. The stretching vibration peak at 1084 cm^−1^ is attributed to an alcoholic hydroxyl group, and the characteristic absorption peak at 954 cm^−1^ is assigned to the methyl group of the quaternary ammonium group [33]. Comparison of the three FTIR spectra of CCPAD with CC and P(AM-DMC) in Figure 2 shows that the absorption peaks of NH stretching vibration at 3409 cm^−1^ and 3194 cm^−1^ are larger because of the superposition of the N-H absorption peaks in CMCS and AM. The absorption peak in the range of 1611.71–1664.75 cm^−1^ is assigned to the stretching vibration absorption peak of C=O produced by the superposition of the C=O absorption peaks in CMCTS and AM. The characteristic absorption peaks of CC and P(AM-DMC) are observed in the FTIR spectrum of CCPAD. This result indicates that the CCPAD was successfully prepared by graft polymerization of CC, AM, and DMC.

#### 3.2.2. ^1^H NMR Characterization

**Figure 3:**
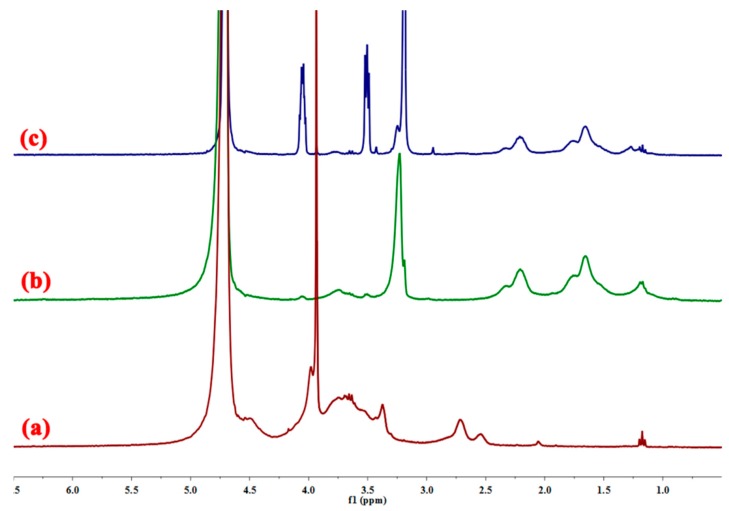
^1^H-NMR spectra of (**a**) CMCS, (**b**) P(AM-DMC), and (**c**) CCPAD.

In Figure 3c, the peak at 1.17–1.27 ppm is assigned to the proton absorption peak of –CH_2_, and the peak at 1.64–1.67 ppm is attributed to the methylene proton of –CH_2_–CH–CONH_2_ [34]. The signal at 2.19–2.22 ppm represents the methyne proton of –CH_2_–CH–CONH_2_, and the peak of 2.92–2.95 ppm is assigned to the proton of NH_2_. The chemical shift at 3.18–3.19 ppm is assigned to the methyl proton of –N–(CH_3_)_3_, the peak of 3.49–3.52 ppm is assigned to the methylene proton of –CH_2_–N–(CH_3_)_3_, and the signal at 3.63–3.79 ppm is assigned to the proton on the sugar ring of CMCS [35]. The signal at 3.93 ppm corresponds to the methine proton of the –CH–OH group in the sugar ring of CMCS, and the signals of 4.04–4.06 ppm are attributed to the methylene proton of –O–CH_2_– of CMCS [36]. In Figure 3a,b, the characteristic proton peaks of the CMCS, AM, and DMC are found in the ^1^H NMR spectra of CCPAD. Thus, the two monomers of AM and DMC are successfully grafted onto CMCS.

#### 3.2.3. XRD Characterization

**Figure 4:**
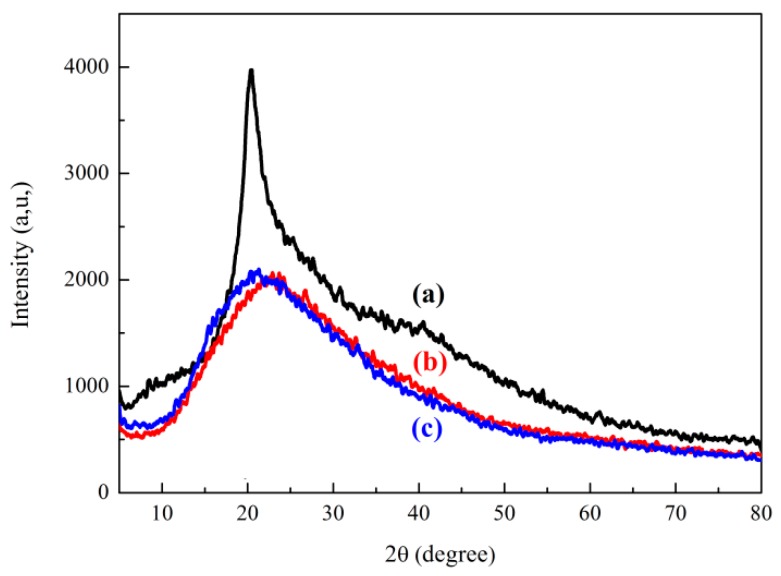
XRD spectra of (**a**) CMCS, (**b**) P(AM-DMC), and (**c**) CCPAD.

In Figure 4a, a narrow and steep diffraction peak appears at 2θ = 20° in the XRD spectrum of CMCS. This peak corresponds to the crystal structure of CMCS. In Figure 4b, a wide and gentle diffraction peak is observed at 2θ = 22° in the XRD spectrum of P(AM-DMC), while a wide and flatter diffraction peak appears near 2θ = 19° in the XRD spectrum of CCPAD. The diffraction peak intensities of P(AM-DMC) and CCPAD are lower than that of CMCS. The hydroxyl and amino groups are distributed on the molecular chain of the CMCS. These groups form intramolecular and intermolecular hydrogen bonds because of the presence of hydrogen bonds and the regularity of the molecules. CMCS tends to locally form tiny crystalline structures [37]. When the three monomers are graft copolymerized, the introduction of AM and DMC will cause the hydrogen bonds of the CMCS molecules to fracture. This process destroys the orderliness of the crystal structure formed, and the overall structural order of CCPAD will be greatly reduced [38]. This result indirectly illustrates the success of the photo-initiated graft polymerization of AM, DMC, and CMCS.

#### 3.2.4. TG-DTG Characterization

**Figure 5:**
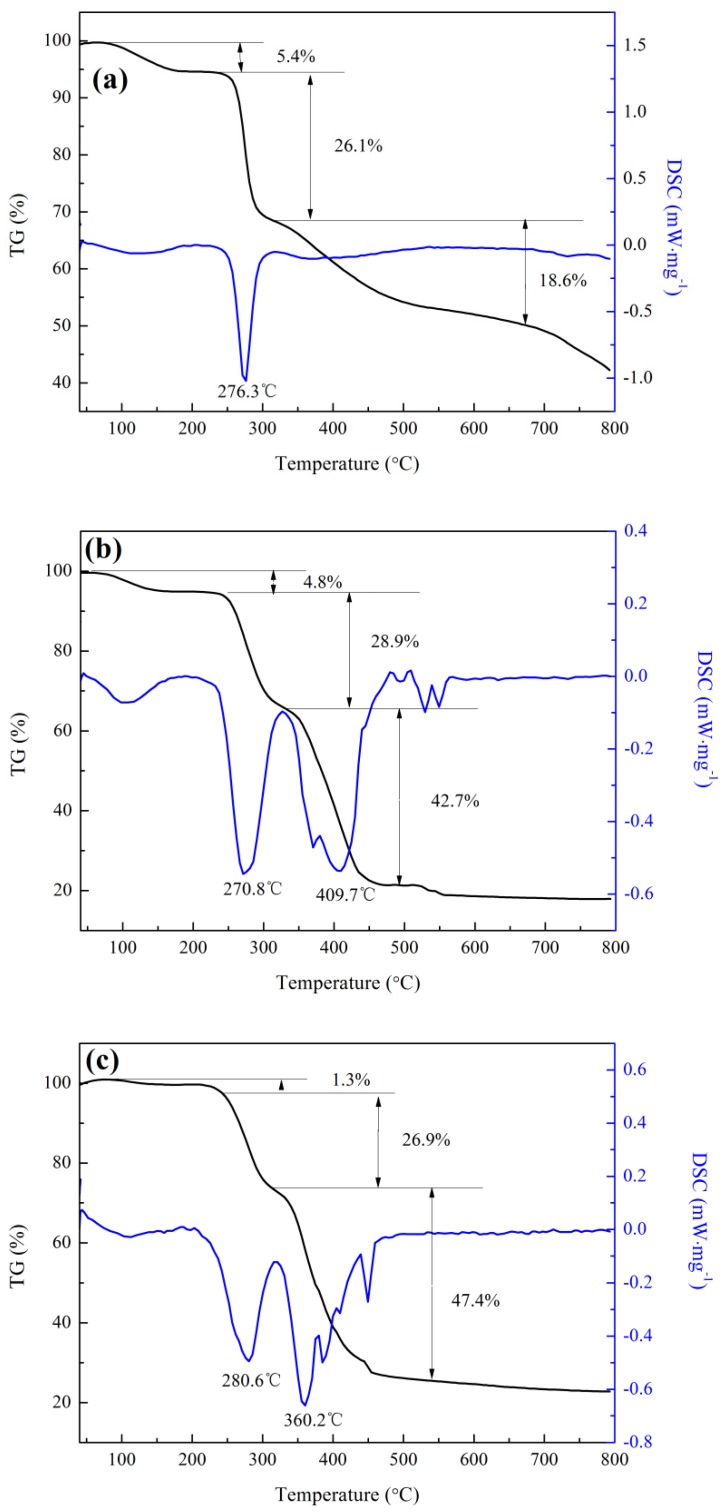
Thermal gravimetric curves of (**a**) CMCS, (**b**) P(AM-DMC), and (**c**) CCPAD.

In Figure 5, the TG-DTG diagram for CCPAD shows three stages of thermal decomposition. The first stage is in the range of 40–200 °C. During this stage, the weight loss rate is 1.27% due to the loss of polymer surface adsorption of water and internal bonding water [39]. The second phase is in the range of 200–330 °C. The weight loss of 26.86% at this phase is due to the imine reaction of the amide group of AM and the demethylation of the quaternary ammonium group of DMC, while the main chain of the CMCS is partially broken and CCPAD begins to decompose [40]. The third stage is in the range of 320–560 °C, and the weight loss is 47.40%. The main chain of CCPAD is broken, and the organic matter is basically decomposed at this time. The residual is 24.47% with thermal decomposition temperature of 275.78–280.60 °C. Comparison of the TG-DTG curves of CMCS, P(AM-DMC), and CCPAD shows that CCPAD has the lowest water content. This characteristic indicates that this substance does not easily absorb moisture and is good for preservation. Graft copolymerization with AM, CMCS, and DMC changes the partial chemical bonds of the monomer molecules and their intra- (or inter-) molecular interactions, resulting in the alteration of the thermostability of CCPAD, as shown by comparing Figure 5c with Figure 5a,b.

### 3.3. Sludge Dewatering Performance

#### 3.3.1. Effect of Cationic Degree

**Figure 6:**
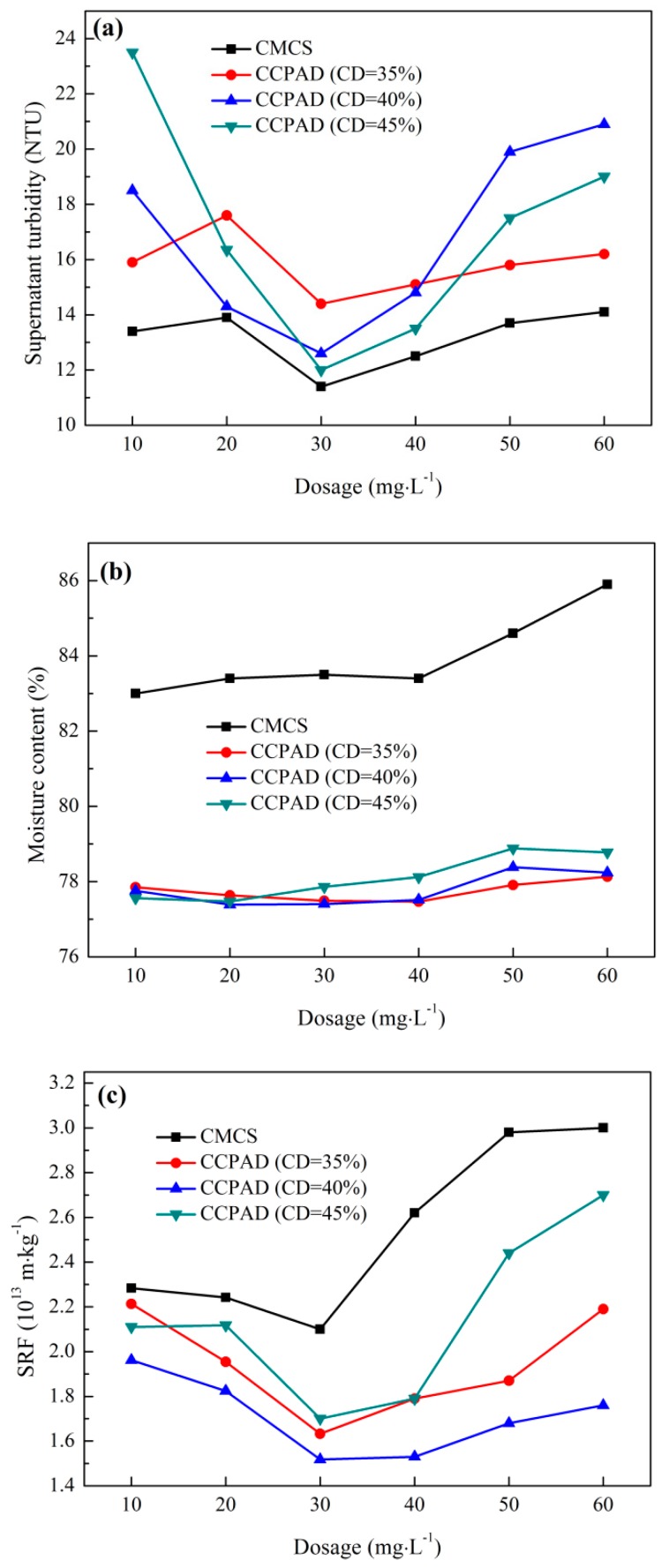

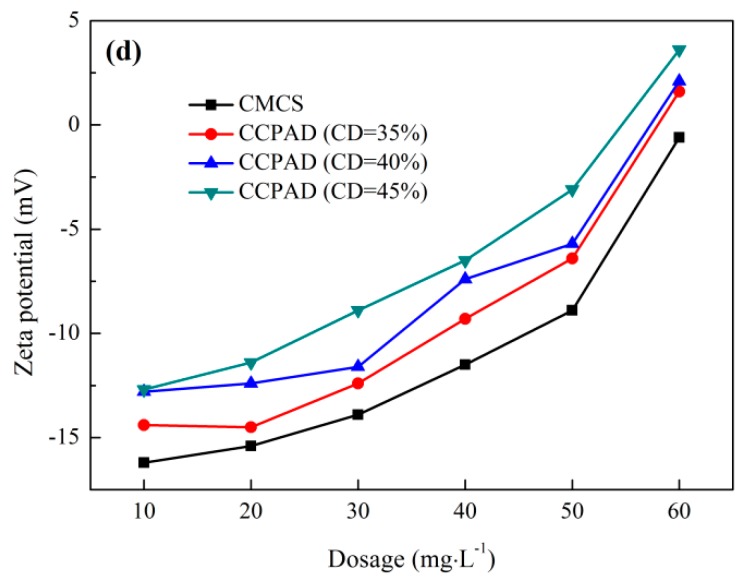
Effect of cationic degree on sludge dewaterability: (**a**) supernatant turbidity, (**b**) moisture content of filter cake, (**c**) SRF, and (**d**) zeta potential.

Sludge specific resistance is a comprehensive indicator of sludge filtration characteristics, and its physical meaning is the resistance of unit mass of sludge to unit filtration area when filtered under a certain pressure. The purpose of this value is used to compare the filtration performance of different sludge. In Figure 6, the sludge specific resistance to filtration and filter cake moisture content decreases first and then increases with increases in the dosage of CCPAD and CMCS. The supernatant turbidity obtained by CMCS is lower than that obtained by CCPAD, while the performance of CCPAD on the moisture content and SRF is better than that of CMCS. The optimal water content of the filter cake by CCPAD is in the range of 20–30 mg/L. The optimal MC and SRF obtained by CCPAD (CD = 40%) at 30 mg/L are 77.3% and 1.5 × 10^13^ m/kg, respectively. In addition, the zeta potential obtained by CCPAD is higher than that by CMCS with increased dosage.

CMCS is a chain structure than can adsorb and bridge sludge particles, while CCPAD also has cationic groups, which can provide adsorption binding sites for negatively charged sludge particles. This characteristic facilitates electricity neutralization, reduction of electrostatic repulsion between the colloidal particles, enlargement of the floccule particles, promotion of aggregation and sedimentation, and improvement of the flocculation performance of the sludge flocs. An appropriate increase in the cationic degree can promote the stretching of the polymer chain, which is favorable for the charge neutralization function [41]. Thus, analysis of the cationic degree from the various indicators of sludge dewatering shows that the appropriate cationic degree is beneficial to improve sludge dewatering performance. This phenomenon is due to the fact that properly increasing the cationic degree of CCPAD at a certain dosage can enhance the electric neutralization ability, and more cationic charges can be provided to neutralize the negative charge on the surface of the sludge particles. In Figure S3, the high cationicity of CCPAD leads to low intrinsic viscosity and short molecular chains, which are not conducive to the adsorption and bridging of sludge particles. Adding an appropriate concentration of CCPAD can not only fully neutralize charge but is also be conducive to the adsorption and bridging between the polymer and sludge particles. Thus, large flocs are formed. When the flocculant amount is small, the positively charged group is not sufficient to neutralize all sludge particles, and the smaller sludge particles can easily clog the filter paper. This phenomenon is not conducive to filtration, resulting in higher MC and SRF [42]. When the amount of flocculant dosage is too large, the sludge particles will be surrounded by positively charged polymers with electrical reversal. This characteristic will cause particles to remain stable because of electrostatic repulsion.

#### 3.3.2. Effect of Intrinsic Viscosity

**Figure 7:**
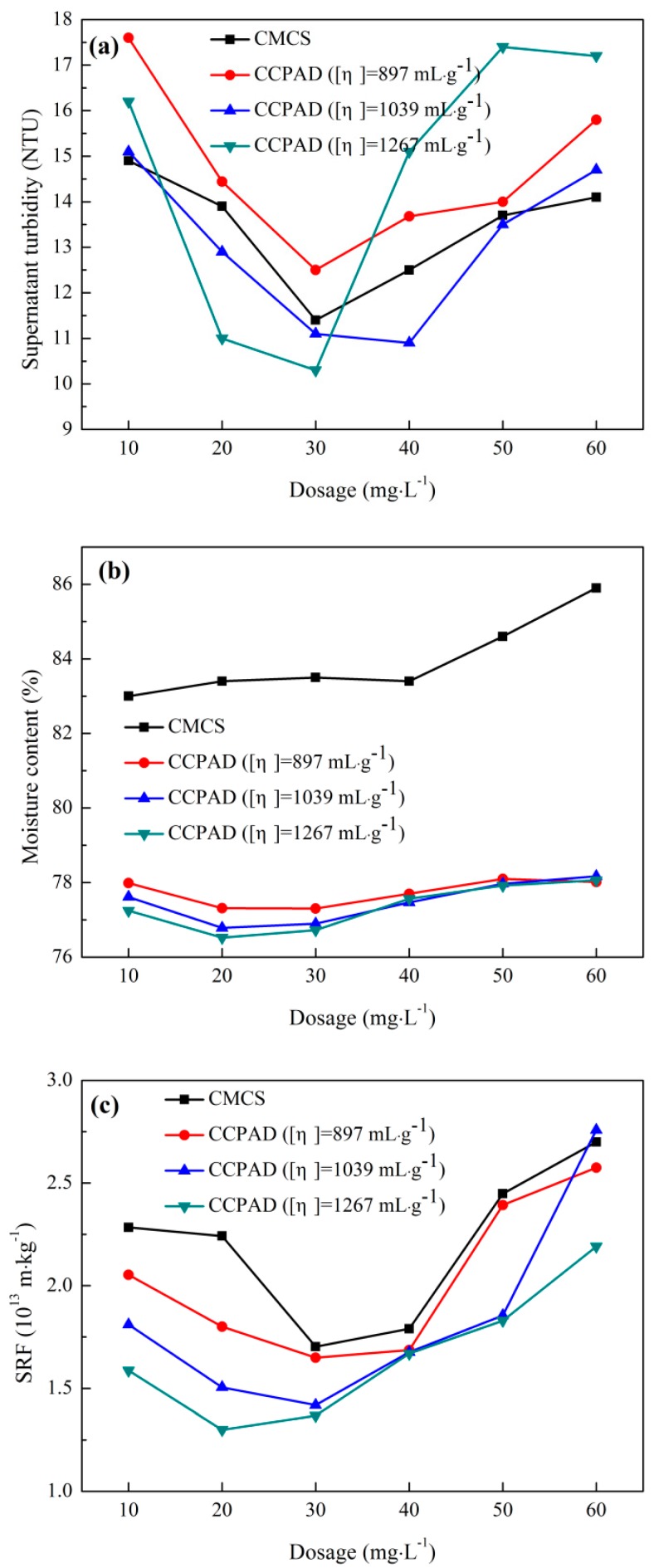
Effect of intrinsic viscosity on sludge dewaterability: (**a**) supernatant turbidity, (**b**) moisture content of filter cake, and (**c**) SRF.

The effect of intrinsic viscosity on sludge dewaterability is shown in Figure 7. With increases in the dosage of CCPAD and CMCS, the moisture content of the filter cake, the supernatant turbidity, and the sludge specific resistance decrease first and then increase. The sludge dewatering performance of CCPAD is better than that of CMCS, because CCPAD has a longer molecular chain presenting positive charges compared with CMCS. The greater intrinsic viscosity will result in better dewatering performance. CCPAD with [η] = 1267 mL/g at 20–30 mg/L has the optimal sludge dewatering performance with supernatant turbidity of 10.3 NTU, MC of 76.5%, and SRF of 1.3 × 10^13^ m/kg.

Based on the flocculation mechanism of cationic flocculants, the cationic CCPAD plays an important role in the destabilization of negatively charged sludge particles mainly because of charge neutralization and adsorption–bridging. The strength of adsorption–bridging depends on the polymer molecular chain length, which is conducive to the full contact of CCPAD with the sludge particles. In adsorption bridges, longer molecular chains can cross and reduce the gaps between colloids to destabilize the colloid particles [43]. The destabilized colloids aggregate with each other and form large flocs that promote aggregation and precipitation. Therefore, at the same dosage, long molecular chains with high intrinsic viscosity increase the probability of colliding CCPAD molecules with colloidal particles. This characteristic enhances the adsorption, bridging function, and sludge dewatering performance. 

When the dosage is too small, the CCPAD molecules cannot completely adsorb the sludge particles, and the positive charge is not sufficient to fully neutralize the sludge particles with poor sludge dewaterability. An appropriately increased dosage can enhance the charge neutralization and adsorption bridging between CCPAD molecules and sludge particles. This process forms large flocs under the adsorption and bridging function. Thus, flocs and water are more easily separated, and a clear supernate is obtained. However, too high a dosage deteriorates the muddy water separation performance of the sludge. The excessive dosing amount causes the neutralized sludge particles to be positively charged and become stable again, and the electrostatic repulsion between the sludge particles is restarted [Figure 5d] [44].

#### 3.3.3. Effect of pH

**Figure 8:**
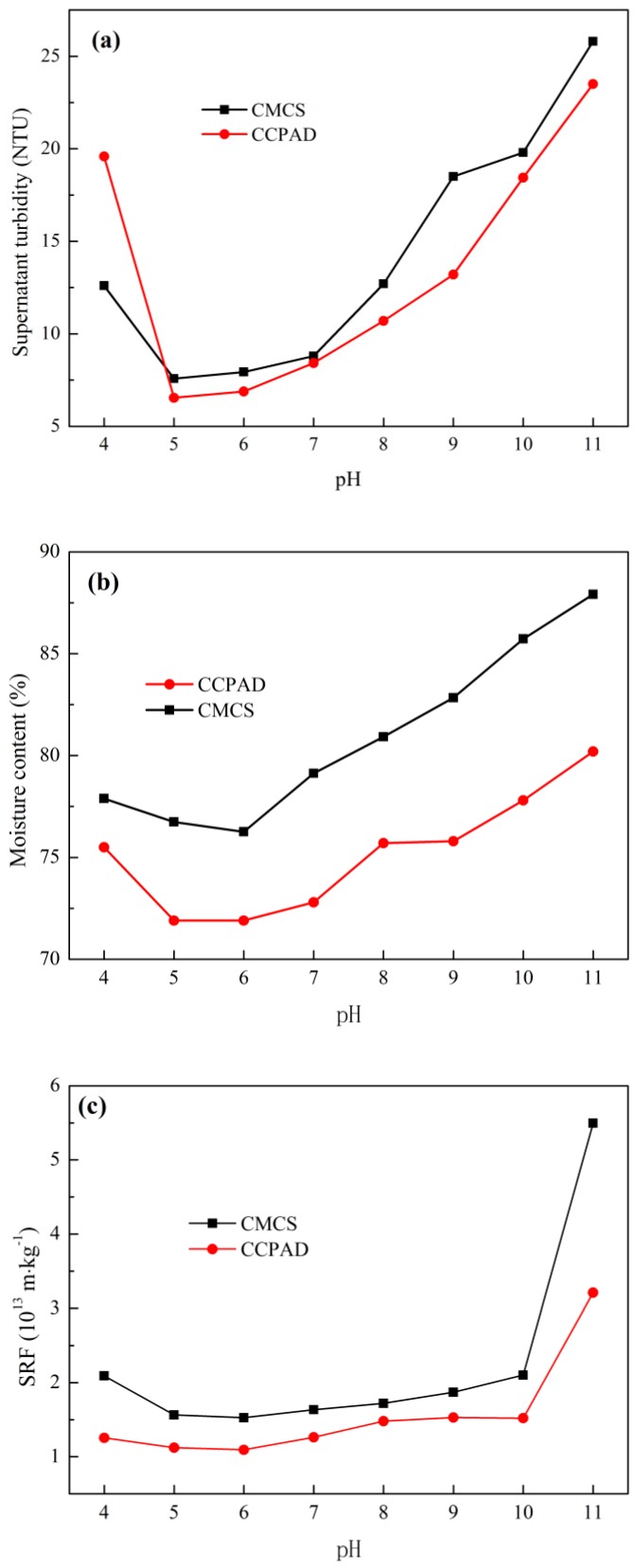

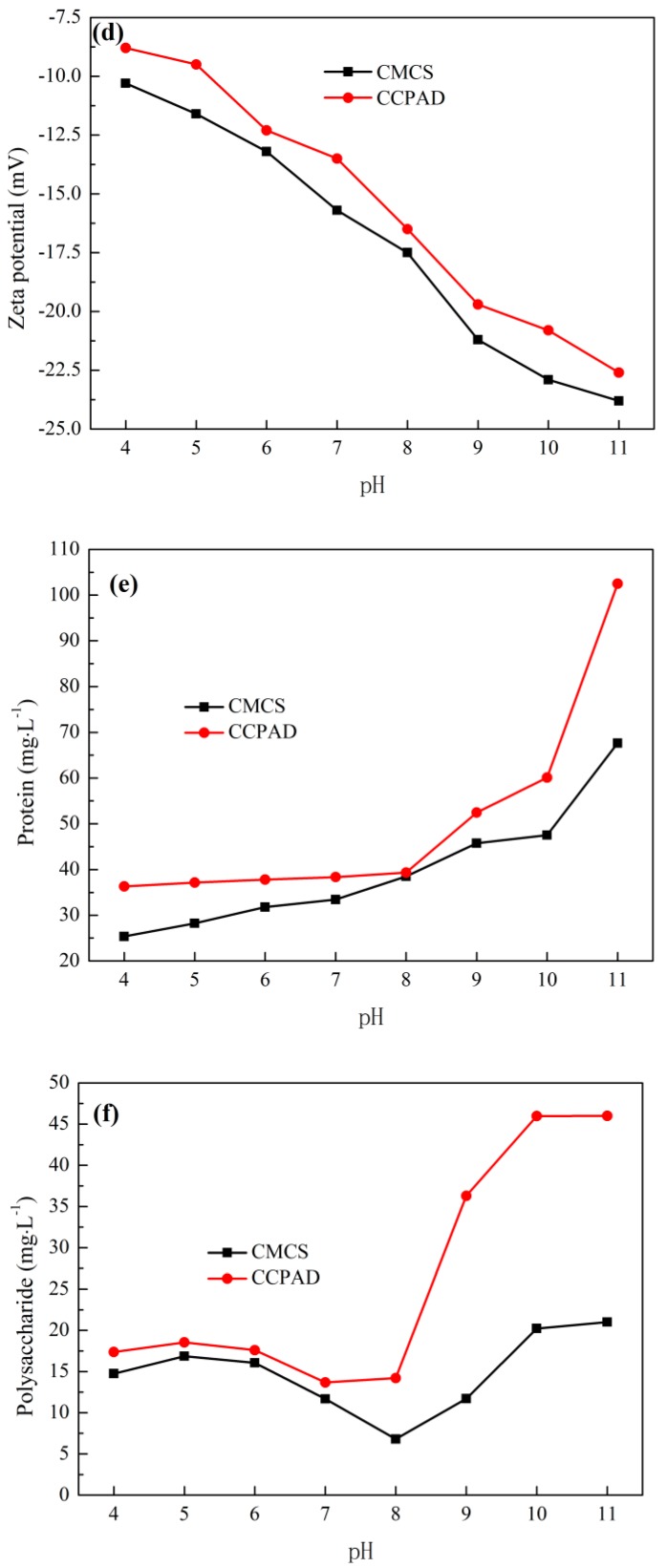
Effect of pH on sludge dewaterability: (**a**) supernatant turbidity, (**b**) moisture content of filter cake, (**c**) SRF, (**d**) zeta potential, (**e**) protein, and (**f**) polysaccharide.

The effect of pH on sludge dewaterability is shown in Figure 8. With increase in the pH value, the moisture content and specific resistance to filtration generally decrease and then increase, and the conditioning performance of CCPAD is better than that of CMCS. At pH 5–6, CCPAD ([η] = 1267 mL/g and CD = 40%) exhibits the best effect on sludge conditioning and dewatering, and the filter cake moisture content, supernatant turbidity, and sludge specific resistance to filtration reach minimum values of 76.26%, 1.09 × 10^13^ m/kg, and 6.54 NTU, respectively. The pH condition will change the surface characteristics of the sludge particles and also has a certain influence on the extracellular polymer in the sludge. In Figure 8b,c, CCPAD shows good conditioning and dewatering performance on the sludge at pH 5–6. Under acidic or alkaline conditions, the activated sludge colloidal particles will be hydrolyzed, and the extracellular polymer in the sludge will be degraded with the release of the internal water from the sludge particles. Large flocs easily form under the action of CCPAD. However, the charge resistance between the sludge particles under alkaline conditions is larger than that under acidic conditions [45].

As the pH increases, the negative zeta potential increases continuously [Figure 8d]. This phenomenon is due to the fact that the high pH value will increase the negative charge density of the colloidal surface. With the adjustment of pH, the sludge particles may adsorb H^+^ or OH^−^ ions, thereby affecting their own chargeability. The effect of pH on the EPS (extracellular polymeric substances) in the supernatant is shown in Figure 8e,f. With the change in pH, the protein and polysaccharide contents in the filtrate change distinctly. The protein increases gradually with the pH value. By contrast, the polysaccharide shows a fluctuating trend, that is, an increase-decrease-increase, and the maximum polysaccharide content is 45.98 mg/L at pH 10. The protein and polysaccharide contents obtained by CCPAD are higher than those obtained by CMCS. Under acidic and alkaline conditions, the structure of the extracellular polymer will break down, making the EPS elute into the flocculated supernatant. This process results in increased levels of polysaccharides and proteins in the sludge supernatant [46]. In addition, the protein and polysaccharide contents under alkaline conditions are higher than those under acidic conditions. Given that the EPS has a negative charge, the electrostatic repulsive force in the alkali increases, making some EPS disperse in the sludge supernatant and increasing the solubility of EPS in the supernatant and the polysaccharides and proteins [47].

#### 3.3.4. Settleability of Sludge after Flocculation

**Figure 9:**
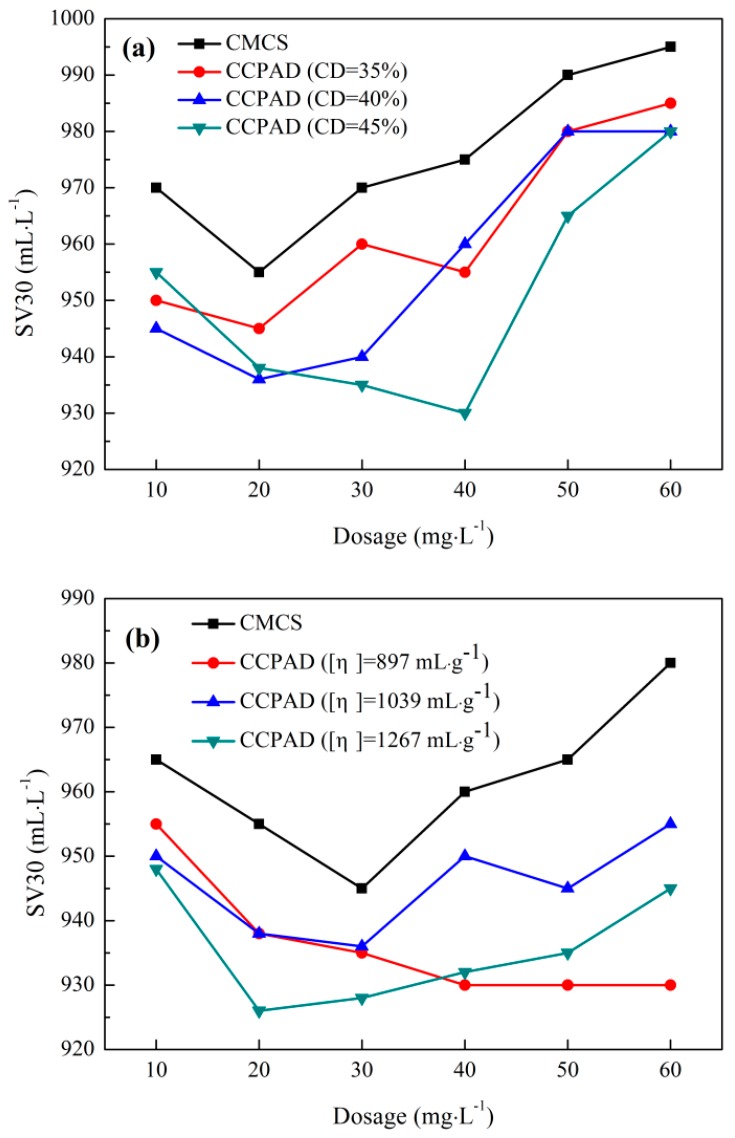

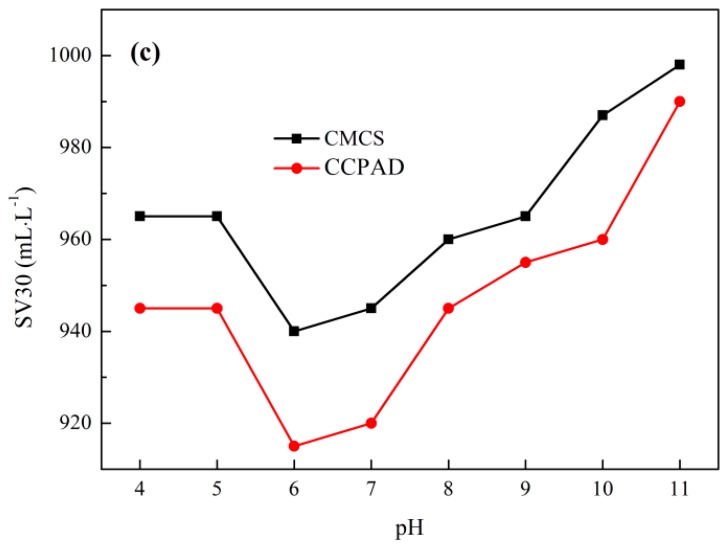
Sludge settleability: (**a**) cationic degree, (**b**) intrinsic viscosity, and (**c**) pH.

The effect of dosage and pH on sludge settling performance for 30 min is shown in Figure 9. The settling performance of the sludge is generally used to study the effect of sludge separation and determine the sludge sedimentation rate. The effect of cationic degree on the settling performance is shown in Figure 9a. Too low and too high cationic degrees are not conducive to the settlement of sludge, and the effect of CCPAD on sludge settling is slightly worse than that of CMCS. The intrinsic viscosities of the three cationic CCPADs are not high, and they mainly show the effect of neutralization and with smaller flocs. In Figure 9b, the higher intrinsic viscosity of CCPAD results in lower sludge sedimentation rate. The highest sludge sedimentation rate is obtained when 20 mg/L CCPAD ([η] = 1267 mL/g) is added. The sedimentation rate is 926 mL/L because of the large intrinsic viscosity, which enhances the adsorption and bridging function. On the basis of electricity neutralization, larger floc particles will more easily form and settle under gravity [48]. From Section 3.3.3, at pH of 5–6, the minimum moisture content of the filter cake and specific resistance of the sludge are obtained, and the supernatant is clear. However, at pH 6–7, the best sedimentation is obtained, and the minimum sedimentation rate is 910 mL/L. Under strong acid and strong alkaline conditions, the sludge particles will release the water inside the particles, and the water content of the sludge will increase. However, due to the effect of the flocculant, the formed flocs will partially float on the water surface, making it difficult to settle [49].

### 3.5. Sludge Morphology

**Figure 10:**
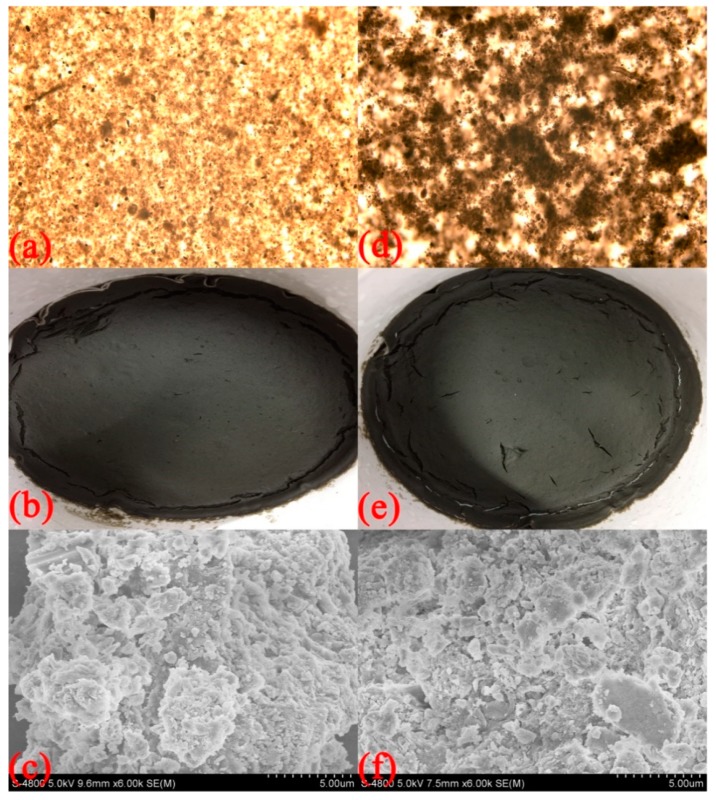
SEM images and photographs of sludge: (**a**–**c**) original sludge, (**d**–**f**) sludge conditioned by CCPAD.

The sludge and sludge cake morphology before and after sludge conditioning are shown in Figure 10. The micrographs show that the unadjusted sludge has small and evenly dispersed sludge particles with a certain consistency, and sludge particle and water are difficult to separate. After CCPAD conditioning, the separation of mud and water is clearly observed. CCPAD enlarges the sludge particles and promotes aggregation through neutralization and adsorption and bridging. This phenomenon can accelerate the separation of sludge and water. The surface of the dewatered sludge cake is relatively unbroken with no cracks. [Figure 10b,e]. After conditioning and dewatering, the mud cake becomes tight and with a noticeable crack. These characteristics show that sludge conditioning by CCPAD can promote the separation of sludge and water, and this process is beneficial to sludge dewatering.

The scanning electron micrographs in Figure 10c,f shows the original sludge particles are small in size with unevenly dispersed arrangement on the surface and strong adhesion to water. After CCPAD conditioning, the number of sludge particles increases, and these particle accumulate with dense structure and smooth surface. This phenomenon is primarily due to the fact that the positively charged CCPAD molecules are electrically neutralized with the negatively charged sludge particles. Moreover, the adsorption and bridging cause the formation of the destabilized particles into large aggregates and flocs [50].

## 4. Conclusions

In this study, CMCS, AM, and DMC were used as raw materials to synthesize chitosan-based flocculant CCPAD through UV-induced polymerization. The optimum synthesis conditions for the photo-initiated graft polymerization of CCPAD by single-factor experiment were determined as follows: total monomer concentration, 40%; CMCS concentration, 8%; cationicity, 40%; photoinitiator concentration, 0.04%; illumination time 2 h; and pH 8. The characterization results show that the successfully synthesized polymer CCPAD had the functional group structure of CMCS, AM, and DMC in the main chain. The sludge condition and sludge dewatering performance of CCPAD were performed and evaluated by supernatant turbidity, moisture content, and specific resistance to filtration. The optimal sludge dewatering performance, with supernatant turbidity of 6.54 NTU, moisture content of 76.26%, and specific resistance to filtration of 1.09 × 10^13^ m/kg, was obtained at pH 5–6 and 20 mg/L CCPAD with intrinsic viscosity of 1267 mg/L. In addition, Zeta potential results show that the main mechanism of sludge flocculation and conditioning was adsorption bridging and charge neutralization. Thus, photopolymerization technology is an efficient and low-cost environmentally friendly initiation technology with good application prospects. It also provides direct evidence for the application of chitosan as a flocculant. Designing and modifying chitosan according to the characteristics of pollutants is the design target of chitosan-based flocculants with high efficiency.

## Figures and Tables

**Table 1:** Sludge properties.

Indices	Parameters
Moisture content	97.9%
pH	7.13
Organic matter content (g/kg)	308.73
Sludge specific resistance to filtration (m/kg)	3.74 × 10^13^
Physical condition	Dark brown with fine particles and a foul stench

**Table 2:** Comparisons of initiation techniques to induce graft-polymerization.

Initiation Methods	Reaction Conditions	Advantages	Disadvantages	References
Photoinitiated polymerization	Normal temperature, normal pressure, reaction time 0.5–2.0 h	Simple operation, a product with high purity, good solubility, fast polymerization speed, environmentally friendly process, energy saving, low production costs.	The initiation mechanism needs further study. Ultraviolet light is easily attenuated in the reaction solution.	[22]
Plasma-initiated polymerization	Normal temperature and pressure, polymerization temperature 10–60 °C, discharge time 0–120 s	No requirement of external initiator, high purity of polymerization product, low cost.	Expensive equipment, Complicated operation. It is still in the laboratory stage with the high investment and requiring vacuum experimental conditions.	[23]
Thermal initiation polymerization	Polymerization temperature 10–100 °C, reaction time 3–24 h	The initiator is thermally decomposed to generate free radicals to initiate polymerization, and the technology is mature and easy to realize industrial production.	Long reaction time with heating, high energy consumption, low product purity, poor solubility.	[24]
Radiation initiated polymerization	Normal temperature and pressure, Radiation time 0–600 s	Low cost, easy operation, uniform reaction, no need to add initiator, fast reaction rate and high product purity; being carried out at room temperature	It is difficult to control the polymerization, and the product is difficult to separate with many residual monomers.	[25]
Microwave initiated polymerization	Normal temperature and pressure, Reaction time 0–10 min	High efficiency, sensitive reaction, uniform molecular weight distribution, fast polymerization rate and short reaction time.	Local overheating, prone to explosion or cross-linking, poor solubility of the product	[26]
Ultrasonic initiated polymerization	Frequency 20 kHz–500 MHz, Reaction time 0–240 min	The short time and efficiency not only accelerate the chemical reaction, increase the reaction yield, shorten the reaction time, but also make it possible to carry out chemical reactions that are difficult or impossible.	The acoustic cavitation effect can produce local high temperature and high pressure, making flocculant prone to explosion or cross-linking, and the product has poor solubility.	[27,28]

## Supplementary Materials

The following are available online at http://www.mdpi.com/2073-4360/11/1/95/s1, Figure S1. Effect of total monomer concentration on the synthesis of CCPAD: (a) intrinsic viscosity and (b) grafting ratio and grafting efficiency; Figure S2. Effect of CMCS concentration on the synthesis of CCPAD: (a) intrinsic viscosity and (b) grafting ratio and grafting efficiency; Figure S3. Effect of cationic degree on the synthesis of CCPAD: (a) intrinsic viscosity and (b) grafting ratio and grafting efficiency; Figure S4. Effect of photo-initiator concentration on the synthesis of CCPAD: (a) intrinsic viscosity and (b) grafting ratio and grafting efficiency; Figure S5. Effect of pH on the synthesis of CCPAD: (a) intrinsic viscosity and (b) grafting ratio and grafting efficiency; Figure S6. Effect of illumination time on the synthesis of CCPAD: (a) intrinsic viscosity and (b) grafting ratio and grafting efficiency.
