# Supplementary material for: Synthesis, Characterization, and Sludge Dewaterability Evaluation of the Chitosan-Based Flocculant CCPAD
Source: Polymers (Basel). 2019 Jan 8;11(1):95. doi: 10.3390/polym11010095 (PMC6402025; doi:10.3390/polym11010095)
Supplement: Supplementary file 1 [file polymers-11-00095-s001.pdf]

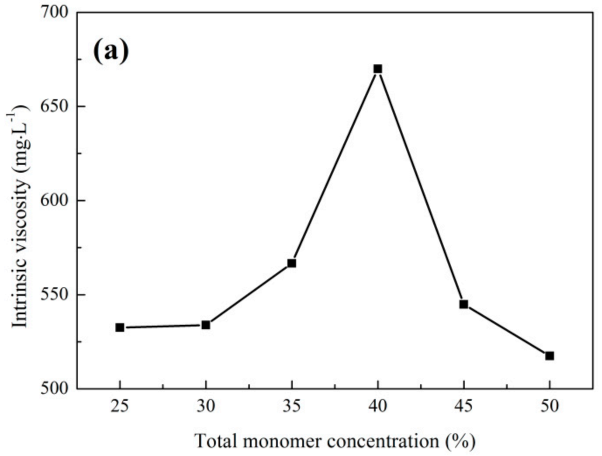

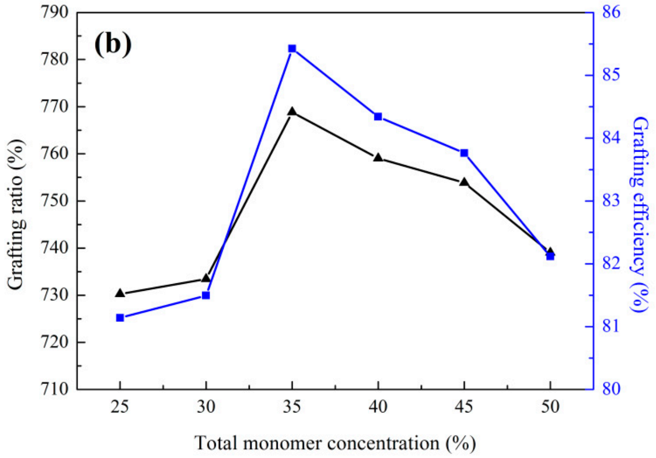

Figure S1. Effect of total monomer concentration on the synthesis of CCPAD: (a) intrinsic viscosity and (b) grafting ratio and grafting efficiency

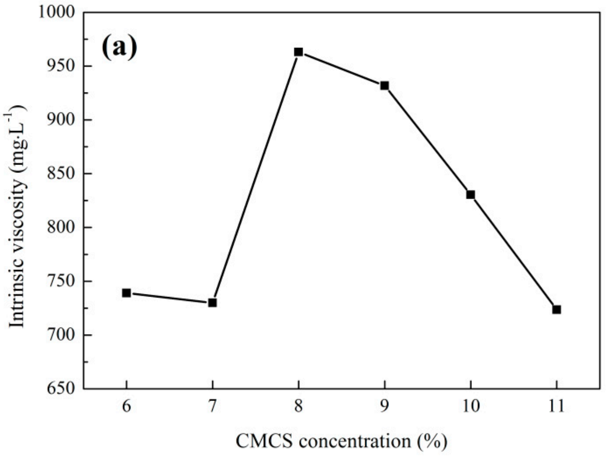

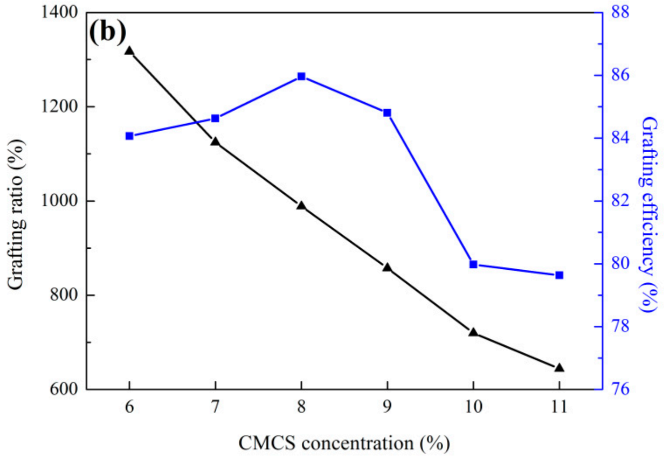

Figure S2. Effect of CMCS concentration on the synthesis of CCPAD: (a) intrinsic viscosity and (b) grafting ratio and grafting efficiency

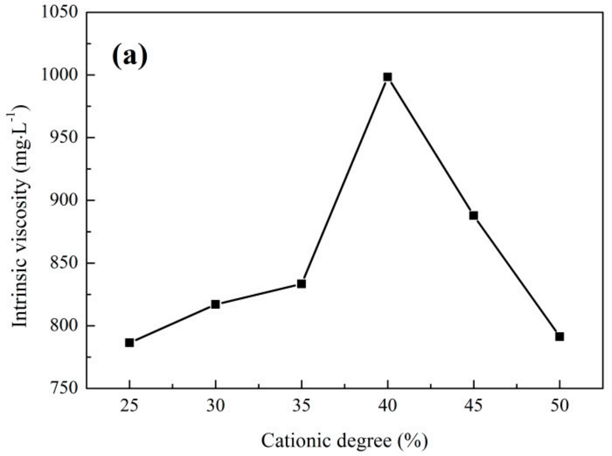

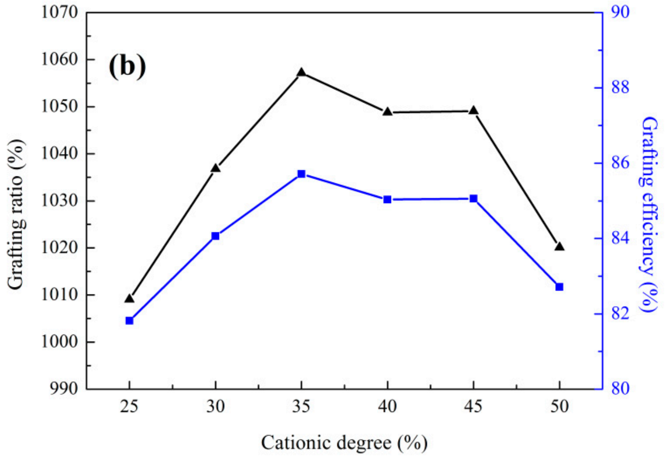

Figure S3. Effect of cationic degree on the synthesis of CCPAD: (a) intrinsic viscosity and (b)

grafting ratio and grafting efficiency

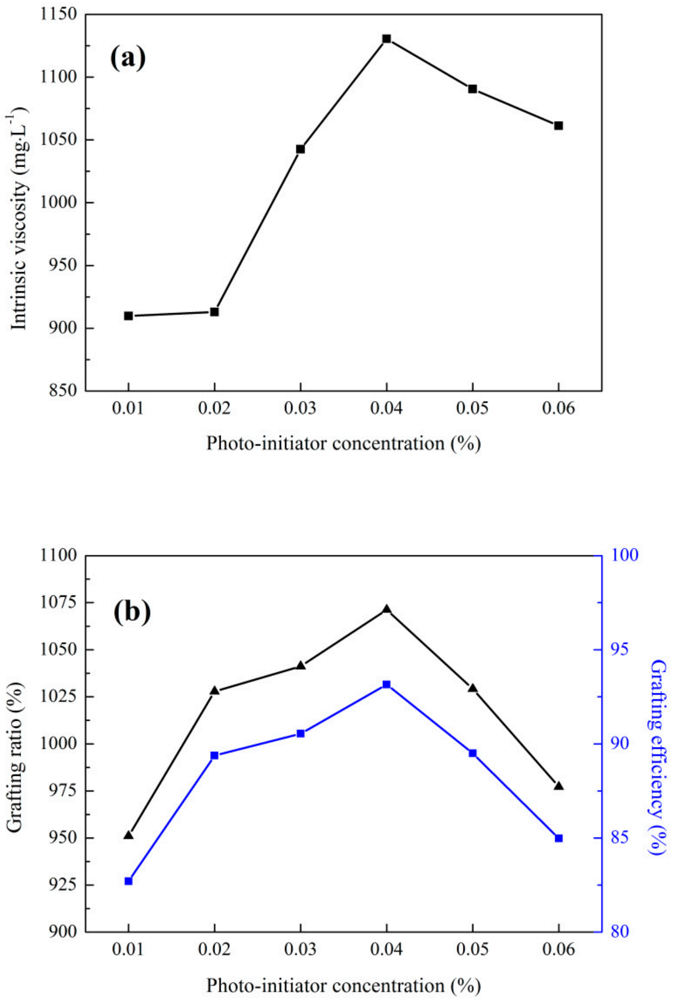

Figure S4. Effect of photo-initiator concentration on the synthesis of CCPAD: (a) intrinsic viscosity and (b) grafting ratio and grafting efficiency

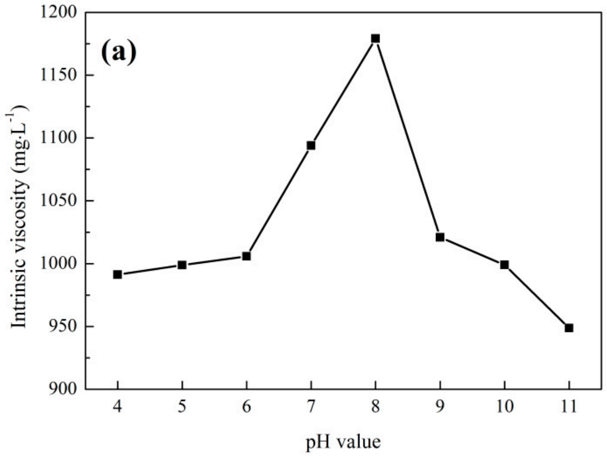

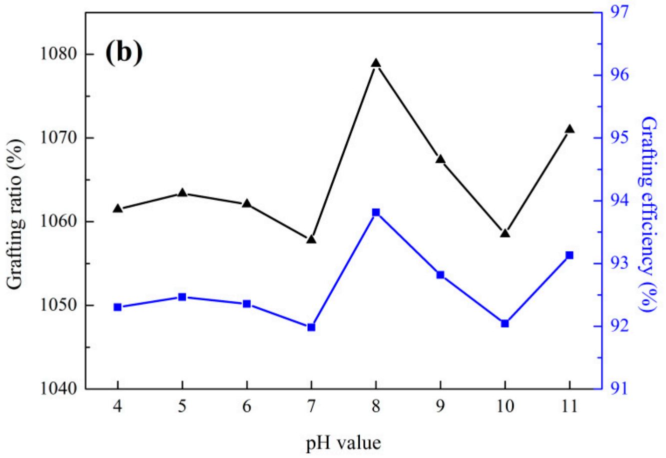

Figure S5. Effect of pH on the synthesis of CCPAD: (a) intrinsic viscosity and (b) grafting ratio and grafting efficiency

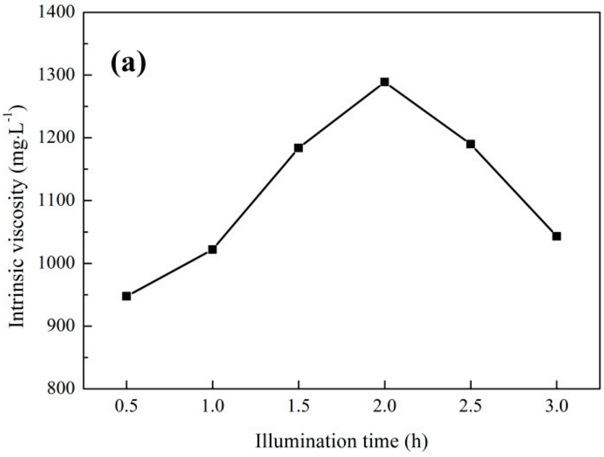

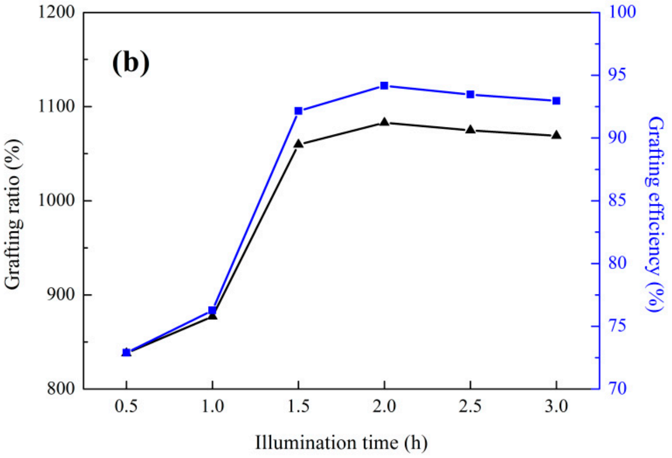

Figure S6. Effect of illumination time on the synthesis of CCPAD: (a) intrinsic viscosity and (b) grafting ratio and grafting efficiency
